# Supplementary figures and images for: A portable lateral flow distance-based paper sensor for drinking water hardness test
Source: PLoS One. 2024 Sep 6;19(9):e0308424. doi: 10.1371/journal.pone.0308424 (PMC11379162; doi:10.1371/journal.pone.0308424)

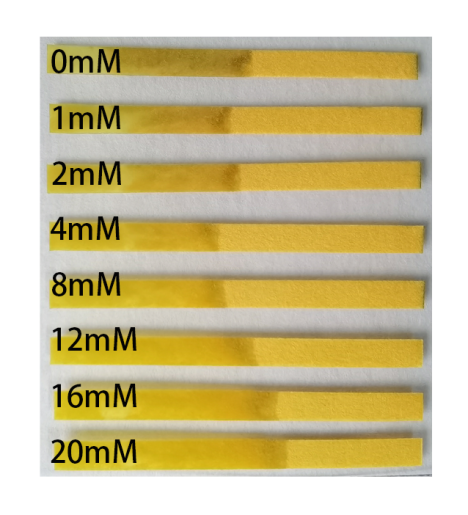

Supplement: S1 Fig — (TIF) [file pone.0308424.s001.tif]

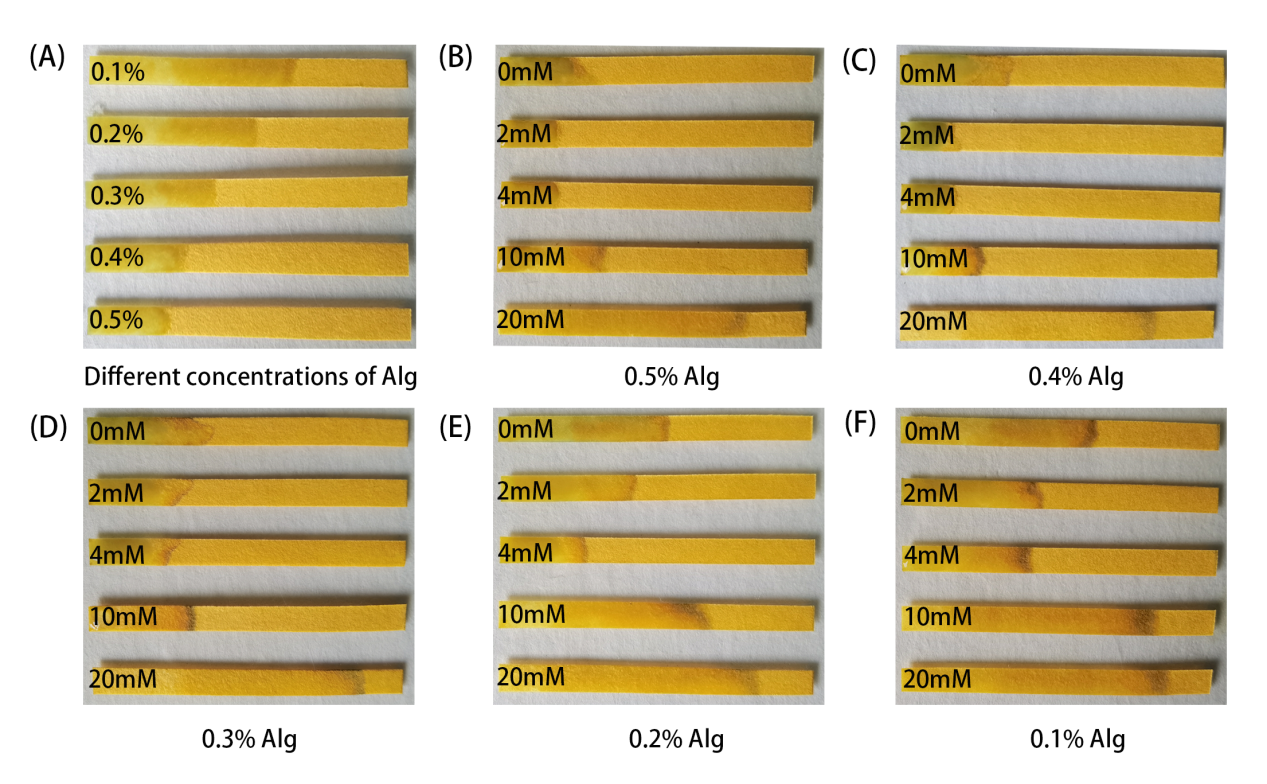

Supplement: S2 Fig — (A) The performance of the sensor towards different concentrations of sodium alginate alone (0.1 wt%, 0.2 wt%, 0.3 wt%, 0.4 wt% and 0.5 wt%), (B-F) the images of the sensors towards the different concentrations of sodium alginate with different concentrations Ca2+ (0, 2, 4, 10 and 20 mmol L-1). (TIF) [file pone.0308424.s002.tif]

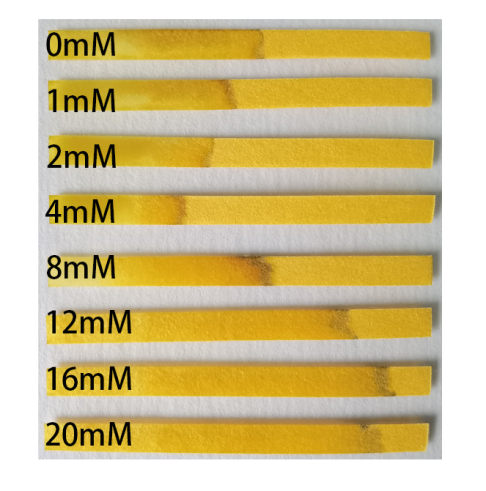

Supplement: S3 Fig — (TIF) [file pone.0308424.s003.tif]

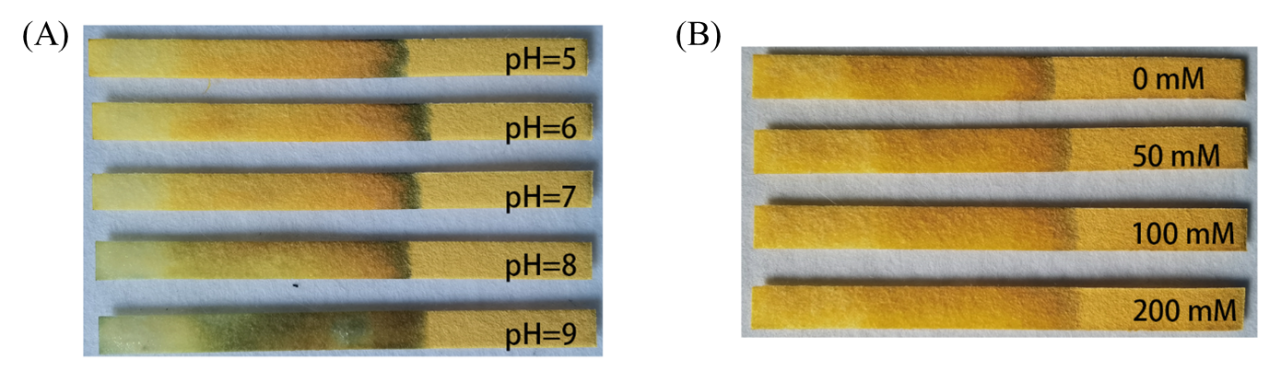

Supplement: S4 Fig — (A) The images of the paper-based sensor towards the aqueous solution with different pH value; (B) The images of the paper-based sensor with the coexistence of different concentrations NaCl (0, 50, 100 and 200 mmol L-1) with Ca2+ (10 mmol L-1), respectively. (TIF) [file pone.0308424.s004.tif]

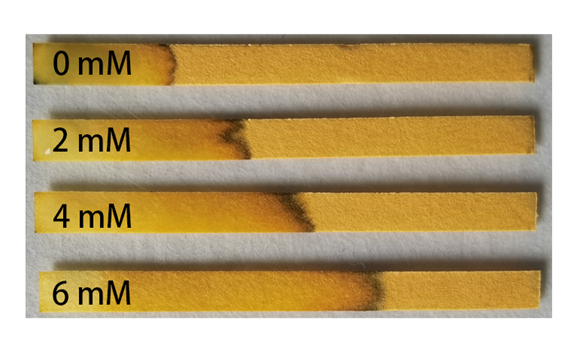

Supplement: S5 Fig — (TIF) [file pone.0308424.s005.tif]
