# Supplementary material for: A portable lateral flow distance-based paper sensor for drinking water hardness test
Source: PLoS One. 2024 Sep 6;19(9):e0308424. doi: 10.1371/journal.pone.0308424 (PMC11379162; doi:10.1371/journal.pone.0308424)
Supplement: S1 Table — (DOCX) [file pone.0308424.s006.docx]

**S1 Table** Comparison of this work with different methods for the detection of Ca^2+^.

| **Method** | **Material** | **Signal mode** | **Detection range** | **Instrument** | **Ref.** |
| --- | --- | --- | --- | --- | --- |
| Electrochemical sensor | AlGaN/GaN transistors | Current | 10^−7^–10^−2^ M | Electrochemical  workstation | S1 |
| Fluorescent sensor | quantum dots | Fluorescence | 5 × 10^−6^ –10^−2^ M | Fluorescence spectrophotometer | S2 |
| Near-infrared sensor | Nanosensor | Fluorescence | 0 - 50 μM | Fluorescence spectrophotometer | S3 |
| DNA-based biosensors | DNAzyme | Gel micrographs | - | Gel electrophoresis | S4 |
| Paper-based sensor | Paper | Distance | 0-14 mM | Without | This work |

**References**

[S1] Asadnia M, Myers M, Umana-Membreno G A, et al. Ca^2+^ detection utilising AlGaN/GaN transistors with ion-selective polymer membranes [J]. Analytica chimica acta, 2017, 987: 105-110.

[S2]Lin Y, Zheng Y, Guo Y, et al. Peptide-functionalized carbon dots for sensitive and selective Ca^2+^ detection [J]. Sensors and Actuators B: Chemical, 2018, 273: 1654-1659.

[S3] Peng L, Yuan G, Ding H, et al. Engineering a near-infrared nanosensor based on supramolecular self-assembly for Ca^2+^ detection and imaging in living cells and mice [J]. Sensors and Actuators B: Chemical, 2021, 332: 129539.

[S4] Yu T, Zhou W, Liu J. Ultrasensitive DNAzyme-based Ca^2+^ detection boosted by ethanol and a solvent-compatible scaffold for aptazyme design [J]. ChemBioChem, 2018, 19(1): 31-36.
