# Supplementary material for: A portable lateral flow distance-based paper sensor for drinking water hardness test
Source: PLoS One. 2024 Sep 6;19(9):e0308424. doi: 10.1371/journal.pone.0308424 (PMC11379162; doi:10.1371/journal.pone.0308424)
Supplement: S2 Table — (DOCX) [file pone.0308424.s007.docx]

**S2 Table** Comparison of this work with different methods for the detection of Mg^2+^.

| **Method** | **Material** | **Signal mode** | **Detection range** | **Instrument** | **Ref.** |
| --- | --- | --- | --- | --- | --- |
| Fluorescent sensor | Organic molecule | Fluorescence | - | Fluorescence spectrophotometer | S5 |
| Electrochemiluminescence sensor | luminol and CdS quantum dots | ECL | 0.01-10 mM | Electrochemical and chemiluminescent analytical system | S6 |
| Fluorescent sensor | Coumarin derivative | Fluorescence | - | Fluorescence spectrophotometer | S7 |
| Electrochemical sensor | Microfluidic chip | Current | 0.1-2 mM | Electrochemical analyze | S8 |
| Paper-based sensor | Paper | Distance | 4-20 mM | Without | This work |

**References**

[S5] Dong Y, Li J, Jiang X, et al. Na^+^ triggered fluorescence sensors for Mg^2+^ detection based on a coumarin salen moiety [J]. Organic letters, 2011, 13(9): 2252-2255.

[S6] Cheng Y, Huang Y, Lei J, et al. Design and biosensing of Mg^2+^-dependent DNAzyme-triggered ratiometric electrochemiluminescence [J]. Analytical chemistry, 2014, 86(10): 5158-5163.

[S7] Gharami S, Sarkar D, Ghosh P, et al. A coumarin based azo-phenol ligand as efficient fluorescent “OFF-ON-OFF” chemosensor for sequential detection of Mg^2+^ and F−: Application in live cell imaging and as molecular logic gate [J]. Sensors and Actuators B: Chemical, 2017, 253: 317-325.

[S8] Gao X, Yu P, Wang Y, et al. Microfluidic chip-based online electrochemical detecting system for continuous and simultaneous monitoring of ascorbate and Mg^2+^ in rat brain [J]. Analytical chemistry, 2013, 85(15): 7599-7605.
