# Supplementary material for: A portable lateral flow distance-based paper sensor for drinking water hardness test
Source: PLoS One. 2024 Sep 6;19(9):e0308424. doi: 10.1371/journal.pone.0308424 (PMC11379162; doi:10.1371/journal.pone.0308424)
Supplement: Attachment — Submitted filename: Response to Reviewers.docx [file pone.0308424.s008.docx]

**Point-by-point responses to editors and reviewers**

We would first like to appreciate the editors and the reviewers for your positive and constructive comments and suggestions, and give us a chance to improve our manuscript.

*Responses to Editor:*

**Question 1:** Please ensure that your manuscript meets PLOS ONE's style requirements, including those for file naming.

**Answer:** Thanks for your careful comments. We have modified our manuscript as PLOS ONE's style requirements.

**Question 2:** We note that the grant information you provided in the‘Funding Information’ and ‘Financial Disclosure’ sections do not match.

**Answer:** Thanks for your friendly suggestion. We have provided the correct grant numbers for the awards for our study in the ‘Funding Information’ section.

Question 3: Please state what role the funders took in the study.

**Answer:** Thanks a lot for your kind suggestion. The funders had no role in study design, data collection and analysis, decision to publish, or preparation of the manuscript. We have included *Role of Funder statement* in our cover letter.

**Question 4:** Please remove any funding-related text from the manuscript and let us know how you would like to update your Funding Statement.

**Answer:** Thank you for your kind reminder. We have removed any funding-related text from the manuscript. We will no longer update our Funding Statement.

**Question 5:** Please ensure that you have an ORCID iD.

**Answer:** Thanks a lot for your kind suggestion. I have an ORCID iD which is 0000-0003-1801-9351.

**Question 6:** Please include a copy of Table 1 which you refer to in your text on page 17 (in PDF format).

**Answer:** Thanks for your careful and kind suggestion. We apologize for our carelessness. We have added Table 1 to the latest version.

**Question 7:** Please include captions for your Supporting Information files at the end of your manuscript, and update any in-text citations to match accordingly

**Answer:** Thanks for your critical comments. We have attached the captions of Supporting Information files at the end of our manuscript, updated any in-text citations to match accordingly.

**Question 8:** Please review your reference list to ensure that it is complete and correct.

**Answer:** Thanks a lot for your kind suggestion. We have carefully reviewed our references list. We ensure that it is complete and correct.

*Responses to Reviewer #1:*

**Question 1:** The abstract section needs to be revised, more information should be mentioned, such as the recoveries of the tested purified water.

**Answer:** Thanks for your professional comments. We have added the recoveries of paper-based sensor into abstract section.

**Question 2**: The third paragraph of Introduction needs to be revised.

**Answer:** Thanks for your professional suggestion which will significantly improve the logic of our article. We have adjusted the order of introduction for paper-based lateral flow sensor , distance signal readout method and analyte-responsive hydrogels.

**Question 3**: “Condition optimization of the paper sensor” section, the author declared that “Moreover, the similar variation tendency was found for the solution and the mixture of Alg and Ca2+, respectively.” The data should be provided.

**Answer:** Thank you for your critical comments. We apologize for our oversight. Originally, we wanted to declared that “the similar variation tendency was found for the solution and the mixture of Alg and Mg2+”, as shown in Fig S1.

**Question 4:** The author declared that “The concentrations of Ca2+ were determined by the paper sensor, the water hardness kit and inductively coupled plasma-mass spectrometry (ICP-MS) (Fig.S5).” However, Fig.S5 is just a graph of the paper-based sensor in response to different added concentration of Ca2+.

**Answer:** Thanks for your careful and kind suggestion. We apologize for our carelessness. Fig S5 is a graph of the paper-based sensor in response to different added concentration of Ca^2+^. We have modified it to “*The concentrations of Ca2+ were determined by the paper sensor (Fig S5), the water hardness kit and inductively coupled plasma-mass spectrometry (ICP-MS).*”

**Question 5**: Table 1 is missing.

**Answer:** Thanks a lot for your careful comment. We have added Table 1 to the latest version.

**Question 6**: In Fig. S2 captions, the concentrations of Ca2+ (0, 1, 2, 5 and 10 mM) is wrong.

**Answer:** Thanks for your careful review. We apologize for our oversight. We have modified the concentration in ***Supporting Information***.

**Question 7**: The discussion of Fig.S4 in the main text is missing. Moreover, other metal ions in the water sample can also affect the sensor's results, not just chloride ions.

**Answer:** Thanks for your professional review. In the main text, we added a brief discussion about the anti-interference capacity of the paper sensor for Ca2+ detection, as shown in Figs 1-4 and Fig S4.

***Responses to Reviewer #2:***

**Question 1**:A thorough literature survey must be included. The characterizations especially for the paper based sensors should be improved. The study related to repeatability and stability must be presented.

**Answer:** Thanks for your professional suggestions.

We made comparison of this work with different methods for the detection of Ca2+ and Mg2+ by a thorough literature survey, which were presented as Tables S1 and S2 in ***Supporting Information***.

In addition, we apologize for missing Table 1 in uploaded manuscript. In Table 1, the RSD of Paper-based sensor ranges from 4.4 to 7.4, which indicates good stability of this method. We have added Table 1 to the latest version.

**Table S1** Comparison of this work with different methods for the detection of Ca^2+^.

| **Method** | **Material** | **Signal mode** | **Detection range** | **Instrument** | **Ref.** |
| --- | --- | --- | --- | --- | --- |
| Electrochemical sensor | AlGaN/GaN transistors | Current | 10^−7^–10^−2^ M | Electrochemical  workstation | S1 |
| Fluorescent sensor | quantum dots | Fluorescence | 5 × 10^−6^ –10^−2^ M | Fluorescence spectrophotometer | S2 |
| Near-infrared sensor | Nanosensor | Fluorescence | 0 - 50 μM | Fluorescence spectrophotometer | S3 |
| DNA-based biosensors | DNAzyme | Gel micrographs | - | Gel electrophoresis | S4 |
| Paper-based sensor | Paper | Distance | 0-14 mM | Without | This work |

**Table S2** Comparison of this work with different methods for the detection of Mg^2+^.

| **Method** | **Material** | **Signal mode** | **Detection range** | **Instrument** | **Ref.** |
| --- | --- | --- | --- | --- | --- |
| Fluorescent sensor | Organic molecule | Fluorescence | - | Fluorescence spectrophotometer | S5 |
| Electrochemiluminescence sensor | luminol and CdS quantum dots | ECL | 0.01-10 mM | Electrochemical and chemiluminescent analytical system | S6 |
| Fluorescent sensor | Coumarin derivative | Fluorescence | - | Fluorescence spectrophotometer | S7 |
| Electrochemical sensor | Microfluidic chip | Current | 0.1-2 mM | Electrochemical analyze | S8 |
| Paper-based sensor | Paper | Distance | 4-20 mM | Without | This work |

**References**

[S1] Asadnia M, Myers M, Umana-Membreno G A, et al. Ca^2+^ detection utilising AlGaN/GaN transistors with ion-selective polymer membranes [J]. Analytica chimica acta, 2017, 987: 105-110.

[S2]Lin Y, Zheng Y, Guo Y, et al. Peptide-functionalized carbon dots for sensitive and selective Ca^2+^ detection [J]. Sensors and Actuators B: Chemical, 2018, 273: 1654-1659.

[S3] Peng L, Yuan G, Ding H, et al. Engineering a near-infrared nanosensor based on supramolecular self-assembly for Ca^2+^ detection and imaging in living cells and mice [J]. Sensors and Actuators B: Chemical, 2021, 332: 129539.

[S4] Yu T, Zhou W, Liu J. Ultrasensitive DNAzyme-based Ca^2+^ detection boosted by ethanol and a solvent-compatible scaffold for aptazyme design [J]. ChemBioChem, 2018, 19(1): 31-36.

[S5] Dong Y, Li J, Jiang X, et al. Na^+^ triggered fluorescence sensors for Mg^2+^ detection based on a coumarin salen moiety [J]. Organic letters, 2011, 13(9): 2252-2255.

[S6] Cheng Y, Huang Y, Lei J, et al. Design and biosensing of Mg^2+^-dependent DNAzyme-triggered ratiometric electrochemiluminescence [J]. Analytical chemistry, 2014, 86(10): 5158-5163.

[S7] Gharami S, Sarkar D, Ghosh P, et al. A coumarin based azo-phenol ligand as efficient fluorescent “OFF-ON-OFF” chemosensor for sequential detection of Mg^2+^ and F−: Application in live cell imaging and as molecular logic gate [J]. Sensors and Actuators B: Chemical, 2017, 253: 317-325.

[S8] Gao X, Yu P, Wang Y, et al. Microfluidic chip-based online electrochemical detecting system for continuous and simultaneous monitoring of ascorbate and Mg^2+^ in rat brain [J]. Analytical chemistry, 2013, 85(15): 7599-7605.

***Responses to Reviewer #3:***

**Question 1**:Review the units. Preferably use mol L-1, g L-1, Temperature in K, etc. According to the international system of units.

**Answer:** Thanks a lot for your kind suggestion. We have reviewed all the units in the manuscript. According to the international system of units, we have converted the units in the manuscript.

**Question 2:** Does study involving pH and temperature variation is necessary for the optimization of sodium alginate concentration?

**Answer:** Thanks for your valuable suggestion. We have tested the anti-interference capacity of the paper sensor for Ca2+ detection including pH and ionic strength. The ionic strength in actual drinking water samples exhibits simple, which rarely contains heavy metal cations. Therefore, we selected common anions for anti-interference detection.

The effect of different temperatures on the formation of calcium oleate has been observed in our previous study. With the increase in temperature, the amount of calcium oleate particles on the wall of the centrifugal tube decreased signiffcantly due to the increase in its solubility. However, the reaction time was largely prolonged if the temperature was too low. Therefore, the temperature of calcium oleate formation was selected as 298K[1].

The corresponding results were represented in ***Results and discussion.***

**Reference**

[1] Xia S, Yin F, Xu L, Zhao B, Wu W, Ma Y, et al. Paper-Based Distance Sensor for the Detection of Lipase via a Phase Separation-Induced Viscosity Change. Anal Chem . 2022; 94(49): 17055-17062.

**Question 3:** Did the authors measure sensor stability?

**Answer:** Thanks for your valuable suggestion. We apologize for missing Table 1 in uploaded manuscript. In Table 1, the RSD of Paper-based sensor ranges from 4.4 to 7.4, which indicates good stability of this method. We have added Table 1 to the latest version.

**Question 4:** If possible, include a table comparing the values obtained with literature data from other sensors.

**Answer:** Thanks a lot for your professional comment. We added Tables S1 and S2 for the comparison of existing sensor, which were attached to ***Supporting Information***.

**Table S1** Comparison of this work with different methods for the detection of Ca^2+^.

| **Method** | **Material** | **Signal mode** | **Detection range** | **Instrument** | **Ref.** |
| --- | --- | --- | --- | --- | --- |
| Electrochemical sensor | AlGaN/GaN transistors | Current | 10^−7^–10^−2^ M | Electrochemical  workstation | S1 |
| Fluorescent sensor | quantum dots | Fluorescence | 5 × 10^−6^ –10^−2^ M | Fluorescence spectrophotometer | S2 |
| Near-infrared sensor | Nanosensor | Fluorescence | 0 - 50 μM | Fluorescence spectrophotometer | S3 |
| DNA-based biosensors | DNAzyme | Gel micrographs | - | Gel electrophoresis | S4 |
| Paper-based sensor | Paper | Distance | 0-14 mM | Without | This work |

**Table S2** Comparison of this work with different methods for the detection of Mg^2+^.

| **Method** | **Material** | **Signal mode** | **Detection range** | **Instrument** | **Ref.** |
| --- | --- | --- | --- | --- | --- |
| Fluorescent sensor | Organic molecule | Fluorescence | - | Fluorescence spectrophotometer | S5 |
| Electrochemiluminescence sensor | luminol and CdS quantum dots | ECL | 0.01-10 mM | Electrochemical and chemiluminescent analytical system | S6 |
| Fluorescent sensor | Coumarin derivative | Fluorescence | - | Fluorescence spectrophotometer | S7 |
| Electrochemical sensor | Microfluidic chip | Current | 0.1-2 mM | Electrochemical analyze | S8 |
| Paper-based sensor | Paper | Distance | 4-20 mM | Without | This work |

**References**

[S1] Asadnia M, Myers M, Umana-Membreno G A, et al. Ca^2+^ detection utilising AlGaN/GaN transistors with ion-selective polymer membranes [J]. Analytica chimica acta, 2017, 987: 105-110.

[S2]Lin Y, Zheng Y, Guo Y, et al. Peptide-functionalized carbon dots for sensitive and selective Ca^2+^ detection [J]. Sensors and Actuators B: Chemical, 2018, 273: 1654-1659.

[S3] Peng L, Yuan G, Ding H, et al. Engineering a near-infrared nanosensor based on supramolecular self-assembly for Ca^2+^ detection and imaging in living cells and mice [J]. Sensors and Actuators B: Chemical, 2021, 332: 129539.

[S4] Yu T, Zhou W, Liu J. Ultrasensitive DNAzyme-based Ca^2+^ detection boosted by ethanol and a solvent-compatible scaffold for aptazyme design [J]. ChemBioChem, 2018, 19(1): 31-36.

[S5] Dong Y, Li J, Jiang X, et al. Na^+^ triggered fluorescence sensors for Mg^2+^ detection based on a coumarin salen moiety [J]. Organic letters, 2011, 13(9): 2252-2255.

[S6] Cheng Y, Huang Y, Lei J, et al. Design and biosensing of Mg^2+^-dependent DNAzyme-triggered ratiometric electrochemiluminescence [J]. Analytical chemistry, 2014, 86(10): 5158-5163.

[S7] Gharami S, Sarkar D, Ghosh P, et al. A coumarin based azo-phenol ligand as efficient fluorescent “OFF-ON-OFF” chemosensor for sequential detection of Mg^2+^ and F−: Application in live cell imaging and as molecular logic gate [J]. Sensors and Actuators B: Chemical, 2017, 253: 317-325.

[S8] Gao X, Yu P, Wang Y, et al. Microfluidic chip-based online electrochemical detecting system for continuous and simultaneous monitoring of ascorbate and Mg^2+^ in rat brain [J]. Analytical chemistry, 2013, 85(15): 7599-7605.
