# Supplementary material for: A portable lateral flow distance-based paper sensor for drinking water hardness test
Source: PLoS One. 2024 Sep 6;19(9):e0308424. doi: 10.1371/journal.pone.0308424 (PMC11379162; doi:10.1371/journal.pone.0308424)
Supplement: Attachment — Submitted filename: Response to Reviewers.docx [file pone.0308424.s009.docx]

**Point-by-point responses to editors and reviewers**

We would like to appreciate the editors and the reviewers for the positive and constructive comments and suggestions, and give us a chance to improve our manuscript.

***Responses to Reviewer #4:***

**Question 1:** There are commercially available instrument for measuring the hardness of water, which like the pH meter. Please also introduce them in the introduction section, and include this type instrument for comparison in the measurement of hardness of water in the experimental section.

**Answer:** Thanks for your careful and kind suggestions. We investigate carefully all the available related instrument. Several methods for measuring the hardness of water have been reported. Among them, atomic absorption spectrometry and complexometric titration are the classic methods. Additionally, the fluorescence method and ionophore-based ion-selective optode also have been used for for monitoring water hardness. The above content has been included in the introduction section. In the study, we employed the commercial kit for Ca^2+^ determination in control group in the experimental section.

**Question 2:** According to the Reviewer #1 of first-round review, there are several errors in the manuscript. Please double check the manuscript.

**Answer:** Thanks for your kind suggestion. We have carefully corrected the errors pointed out by *the Reviewer #1*. Meanwhile, we checked the manuscript carefully.

**Question 3:** Do not use non-English terms such as "anti-interference" or "foreign ions". Make use of correct terms "Selectivity" and "potentially interfering ions", respectively.

**Answer:** Thanks for your professional suggestion which will significantly improve the readability of our article. We have adjusted non-English terms according to your advice .

**Question 4:** Please avoid using first-person narratives for a scientific paper.

**Answer:** Thanks for your careful and kind suggestion. We have switched from first-person narratives to third-person narratives to present scientific facts.

**Question 5:** The shortcomings of present work can be emphasized in the conclusion section.

**Answer:** Thanks for your professional comments. We have emphasized the shortcomings of present work in the conclusion section.

**Question 6:** Please carefully check and unify the units. For example, both the “mM” and “mmol-1” have been used in the manuscript.

**Answer:** Thank you for your critical comments. We apologize for our oversight. We have carefully checked and unified the units as mmol L^-1^.

***Responses to Reviewer #5***

**Question 1:** The English language throughout the manuscript must be revised. There are many typos (e.g. thrice) and strange sentences (e.g. Drinking water quality is a problem concerned by people). The peer-reviewing is not a proof reading service, so the English must be revised by someone dedicated to it.

Answer: Thanks for your kind suggestion. We have carefully revised the English language throughout the manuscript.

**Question 2:** A broader literature revision must be done. There are, at least, two papers dealing with the determination of water hardness using paper-based devices that were not mentioned (10.1016/j.measurement.2020.108085 and 10.3390/su14063363). It should be inserted and discussed.

Answer: Thank you for your valuable suggestion. We have unified and updated the format of the literature throughly. Meanwhile, we have added the mentioned literatures (10.1016/j.measurement.2020.108085 and 10.3390/su14063363) with discussion in the manuscript.

**Question 3:** Eriochrome T is the most usual "reagent" for the determination of water hardness. Why your procedure is better?

Answer: Thanks for your professional comments. Eriochrome T is the most usual "reagent" for the determination of water hardness. In this study, the commercial kit for Ca2^+^ determination employed in the control group is made up of Eriochrome T. Both approaches have advantages and disadvantages. Our procedure provides a portable approach for semi-quantitative detection of drinking water hardness with convenience and low cost. However, the detection of water samples in complex environments needs to be further optimized.

**Question 4:** "anti-interference" sounds strange.

Answer: Thank you for your critical comments. We apologize for using non-English terms. We have replaced it with “Selectivity”.

**Question 5:** The "*C*r values" is not clear how to calculate. Please clarify it.

Answer: Thanks a lot for your suggestions. The calculation of the coverage ratios of water mark was illustrated as below:

P_mark_ = the pixel values of the watermark area= 58765.

P_total_= the pixel values of the whole test strip = 101797

Therefore, *C*r = P_mark_ / P_total_ =58765/101797 = 0.577

**Question 6:** The images were taken with no illumination control? Is the results affected by different illumination conditions? It should be discussed.

Answer: Thanks for your professional comments. In this study, the photographs were taken at ambient light conditions. Moreover, the detection relies on measuring the water flow distance. It is not be affected by the illumination conditions. This is an advantage of the distance sensor.

**Question 7:** Test-t should be performed to compare the results obtained by the proposed method with the reference ones.

Answer: Thanks for your professional comments. We performed the test-t analysis between the proposed paper-based method and the ICP-MS method which is the gold standard for metal content measurement. The p values were calculated as 0.317, 0.353, and 0.596 respectively, corresponding to the marked samples with Ca^2+^ concentrations of 2, 4, and 6 mM, respectively. No significant difference was observed, which demonstrate the reliability of the paper-based method.
